# bMSAF is a prognostic predictor for advanced hepatocellular carcinoma patients treated with immune checkpoint inhibitor camrelizumab and anti‐angiogenic agent apatinib combination therapy

**DOI:** 10.1002/ctm2.1086

**Published:** 2022-10-17

**Authors:** Gehan Xu, Liang Cui, Jin Li, Quanren Wang, Pansong Li, Xuefeng Xia, Xin Yi, Quanlin Guan, Jianming Xu

**Affiliations:** ^1^ The First Clinical Medical College of Lanzhou University Lanzhou China; ^2^ Geneplus‐Beijing Institute Beijing China; ^3^ Jiangsu Hengrui Medicine Co. Ltd. Jiangsu China; ^4^ Department of Oncology Surgery The First Hospital of Lanzhou University Lanzhou China; ^5^ Department of Gastrointestinal Oncology The Fifth Medical Center Chinese PLA General Hospital Beijing China

**Keywords:** angiogenic inhibitors, biomarker, hepatocellular carcinoma, immune checkpoint inhibitors, prognosis

Dear Editor

Hepatocellular carcinoma (HCC) has a high mortality rate worldwide.^1^ Immune checkpoint inhibitors (ICIs) alone^2^ or in combination with anti‐angiogenic drugs^3^, ^4^ have made breakthroughs in the treatment of advanced HCC, but only a minority of patients benefit from these therapies, lacking reliable response predictors. Herein, a targeted panel of 1021 genes screened against 107 blood samples and whole‐exome sequencing (WES) performed for 44 liver tumour tissues were used to identify potential biomarkers for ICI combined with anti‐angiogenic agent treatment.

In total, 118 advanced HCC patients treated with camrelizumab plus apatinib were enrolled from our Phase II RESCUE trial.^4^ Supplementary Table S1 showed the detailed clinicopathological information about patients (*N* = 118), which was similar to the patient characteristics of RESCUE trial (*N* = 190) (Supplementary Table S2).

WES was performed on 44 tumour tissues and paired blood samples. The average sequencing depth was 552× and 99.61% of target sequences were sequenced to at least 10× depth in tumours (Supplementary Table S3). A total of 3560 somatic mutations involving 2886 genes were detected (Supplementary Table S4). Analyses of tissue‐based biomarkers exhibited that tissue‐based tumour mutation burden (tTMB) (optimal cutoff = 52, Supplementary Figure S1A–D) and tumour neoantigen burden (TNB) (optimal cutoff = 26, Supplementary Table S5, Supplementary Figure S2A–D) were not apparent association with response rate or survival benefit in the camrelizumab + apatinib, in which patients with high tTMB showed a non‐significant trend of longer PFS (*p* = .063) compared with those with low. This was consistent with previous study,^5^ suggesting that there may be no relationship between CD8^+^ T cell levels and neoantigen load in HCC.^6^ Besides, PD‐L1 status was not significantly associated with survival or clinical efficacy in 54 HCC tissues (Supplementary Figure S3A–C). Collectively, all tested tissue‐based tumour biomarkers failed to predict prognosis and response resulting from the combination therapy. Because of the small size for available tissue samples, above findings might require further validation.

Subsequently, we sequenced circulating tumour DNA (ctDNA) of 107 baseline blood samples. The average sequencing depth was 1819× and 99.96% of target sequences were sequenced to at least 10× depth (Supplementary Table S6). A total of 594 somatic mutations involving 254 genes were obtained (Supplementary Table S7). The bTMB was associated with age and alpha‐fetoprotein (AFP) (Supplementary Table S8). In total patients, low bTMB (bTMB‐L, optimal cutoff = 4) showed significantly longer OS (*p* = .019) compared with high bTMB (bTMB‐H) (Figure 1A and B), and bTMB was significantly associated with disease control rate (DCR) (Figure 1C and D), but not objective response rate (ORR) (Supplementary Figure S4A). Furthermore, bTMB also significantly affected PFS of first‐line patients and DCR of second‐line patients (Supplementary Figure S5A–F).

**FIGURE 1:**
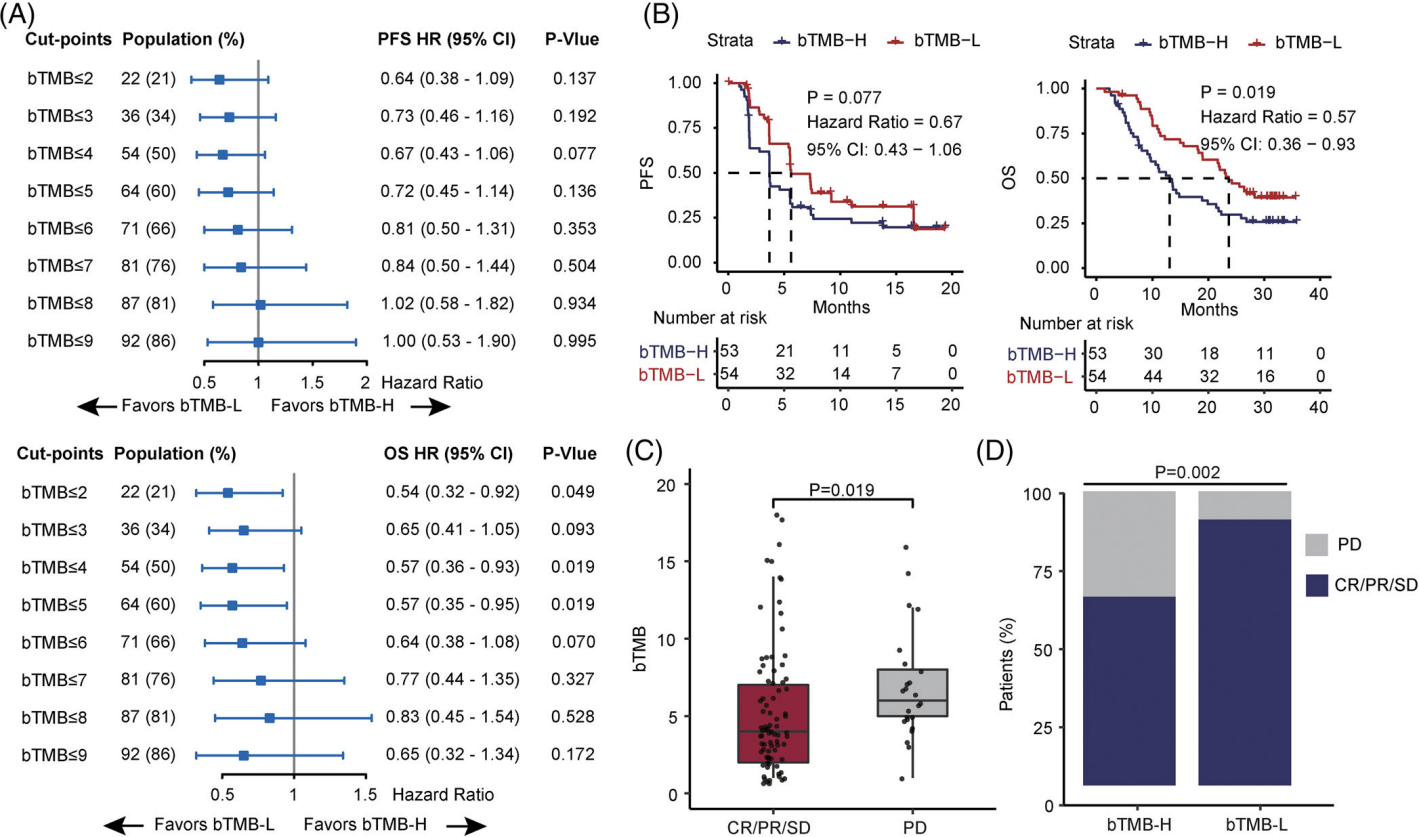
Low bTMB is associated with better clinical benefit from camrelizumab plus apatinib combination therapy. (A) Forest plots of HRs of PFS and OS comparing patients at varying bTMB cutpoints. (B) Kaplan‐Meier analysis of PFS and OS in patients with bTMB‐L (bTMB ≤4) and bTMB‐H (bTMB > 4). (C) bTMB in patients with CR/PR/SD (*n* = 81) versus those with PD (*n* = 24) (median: 4 vs. 6 mutations, Wilcoxon *p* = .019). (D) Disease control in patients with bTMB‐H versus those with bTMB‐L (DCR: 64% versus 90%, Fisher's exact *p* = .002)

Because above findings contrasted with the lack of tTMB predictive effect, we explored the consistency of mutations in 36 paired blood‐tissue samples. In the same genomic regions of WES and target panel, the top 20 mutant genes were identical in blood and tissue (Supplementary Figure S6A). Of the 216 mutations detected in blood ctDNA and tissue DNA, 147 (68.1%) variants were shared by both, 42 (19.4%) variants were unique to blood samples, and the other 27 mutations (12.5%) were private to tissue samples (Supplementary Figure S6B). The bTMB determined by 1021 panel remarkably correlated with tTMB determined by WES (*r* = 0.41, *p* = .012) (Supplementary Figure S6C).

Blood‐based maximum somatic allele frequency (bMSAF) can estimate the ctDNA amount in peripheral blood samples,^7^ thus we also investigated the predictive value of bMSAF. The bMSAF was related to vascular invasion and AFP (Supplementary Table S8). Low bMSAF (bMSAF‐L, optimal cutoff = 0.027, Figure 2A) significantly prolonged PFS (*p* = .004) and OS (*p* = .002) (Figure 2B), which may be attributed to higher bMSAF reflecting a higher tumour burden, leading to immune response suppression.^8^ Moreover, DCR group appeared to be a lower median bMSAF (Figure 2C), and bMSAF‐L group had higher DCR compared with high bMSAF (bMSAF‐H) group (90% vs. 72%, *p* = .043) (Figure 2D), but there was no significant difference between responders and non‐responders (Supplementary Figure S4B). The impact of bMSAF on survival and response rate was not significant in patients treated with first‐line therapy, but it is worth mentioning that the association between bMSAF and PFS was close to significant (Supplementary Figure S7A–C). And bMSAF could significantly distinguish survival benefits of patients treated with second‐line therapy (Supplementary Figure S7D–F).

**FIGURE 2:**
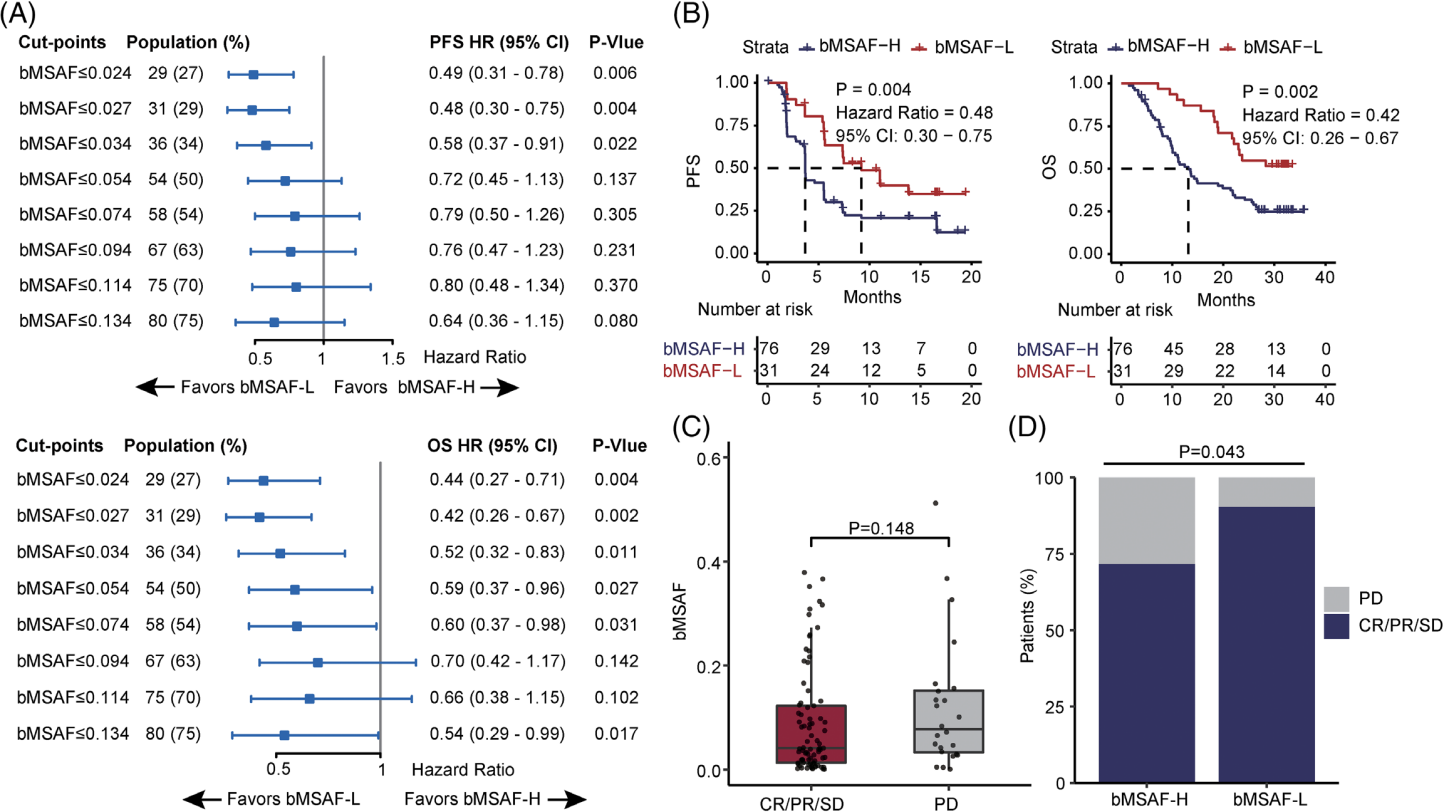
bMSAF better predicts the response to camrelizumab plus apatinib. (A) Forest plots of HRs of PFS and OS comparing patients at varying bMSAF cutpoints. (B) Kaplan‐Meier analysis of PFS and OS in patients with bMSAF‐L (bMSAF ≤0.027) and bMSAF‐H (bMSAF > 0.027). (C) bMSAF in patients with CR/PR/SD (*n* = 81) vs. those with PD (*n* = 24) (median: 0.046 vs. 0.082, Wilcoxon *p* = .148). (D) Disease control in patients with bMSAF‐H versus those with bMSAF‐L (DCR: 72% versus 90%, Fisher's exact *p* = .043)

Next, a remarkable correlation was observed between bTMB and bMSAF (*p* < .001, *r* = 0.50) (Figure 3A). When stratifying patients using 0.04 as the bMSAF cutoff, this correlation was the strongest in patients with bMSAF‐L ≤0.04 (*N* = 42, 39%) (*p* < .001, *r* = 0.62) (Figure 3B and C), where survival in bMSAF‐L or bTMB‐L group was still better (Figure 3D). When bMSAF > 0.04 (*N* = 65, 61%), bMSAF weakly correlated with bTMB (*r* = 0.32, *p* = .009) (Supplementary Figure S8A). Neither bTMB nor bMSAF could significantly distinguish patients with clinical benefit (Figure 3E). We sought to identify patients with prolonged survival in the bMSAF > 0.04 group by combining bTMB and bMSAF, but this combined index did not improve the predictive ability for prognosis compared with bMSAF alone (Supplementary Figure S8B). In view of the above findings, we further determine the relationship among bTMB, bMSAF and prognosis using multivariate Cox analysis and demonstrated that bMSAF independently affected PFS whether in total population (*p* = .023) (Table 1), first‐line patients (*p* = .038) (Supplementary Table S9) and second‐line patients (*p* = .024) (Supplementary Table S10).

**FIGURE 3:**
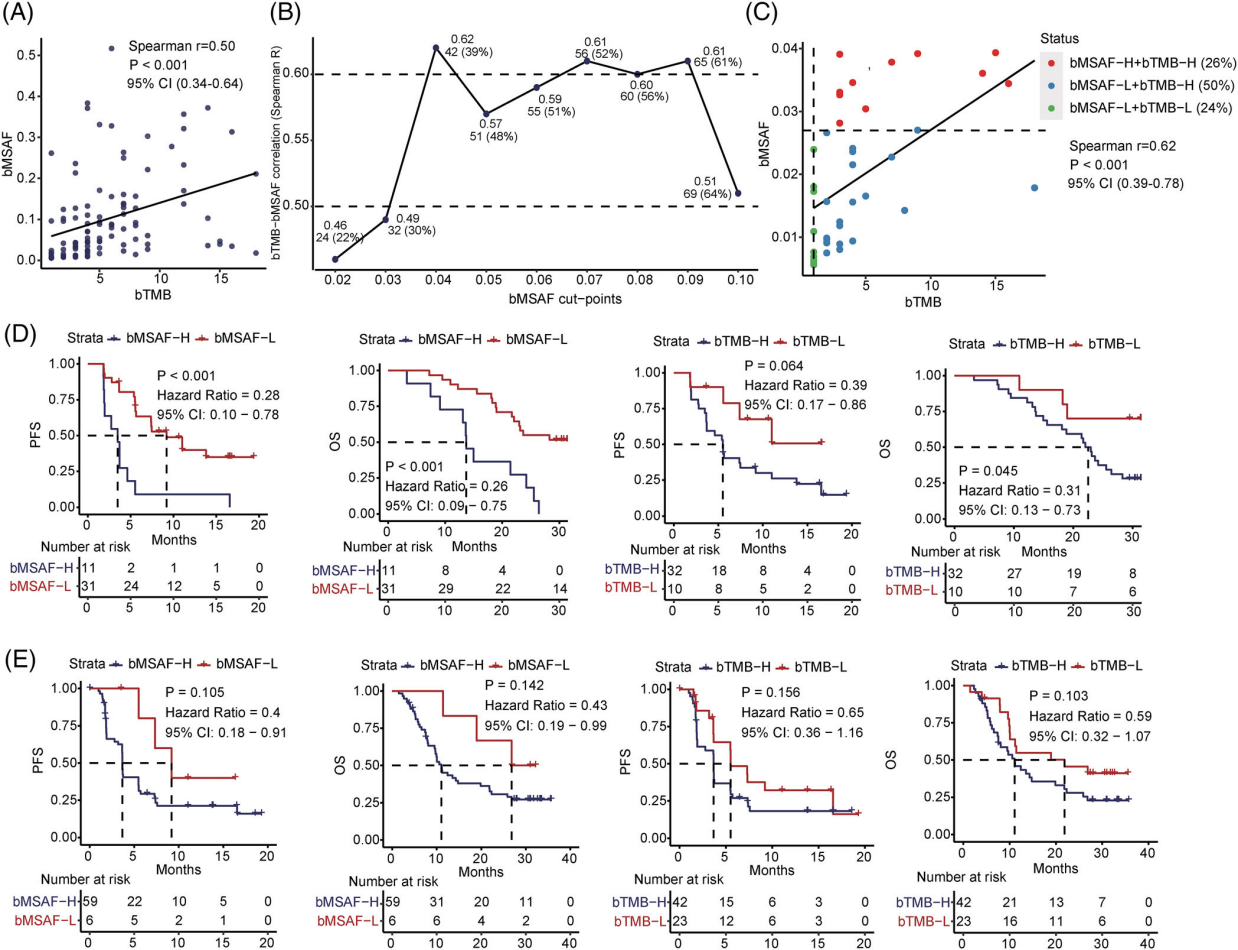
Association between bTMB and bMSAF. (A) Correlation between entire bTMB and entire bMSAF (Spearman's *r* = .50, *p* < .001). (B) Spearman's correlation of bTMB and bMSAF at varying bMSAF cutpoints. Each point is also represented by the corresponding the number of patients (%). When bMSAF ≤4%, the correlation between bTMB and bMSAF is the strongest. (C) High correlation between bTMB and bMSAF in patients with ctDNA MSAF ≤4% (Spearman's *r* = .62, *p* < .001). Patients with bMSAF‐H and bTMB‐H (*n* = 11) are indicated in red, patients with bMSAF‐L and bTMB‐H (*n* = 21) are indicated in blue, and those with bMSAF‐L and bTMB‐L (*n* = 10) are indicated in green. (D) Survival curve analysis of bMSAF and bTMB in patients with bMSAF ≤4% (optimal cutoff: bMSAF = 0.027, bTMB = 1). (E) Survival curve analysis of bMSAF and bTMB in patients with bMSAF > 4% (optimal cutoff: bMSAF = 0.046, bTMB = 4)

**TABLE 1:** Univariate and multivariate analyses of bMSAF on PFS and OS using important clinical characteristics and bTMB as covariates in total patients

**Characteristics**	**PFS**				**OS**			
	Univariate	*p* Value	Multivariate	*p* Value	Univariate	*p* Value	Multivariate	*p* Value
**Age, years (≥60 vs. < 60)**	0.50 (0.28–0.92)	.027	0.44 (0.24–0.83)	.012	0.60 (0.32–1.12)	.110		
**Sex (male vs. female)**	2.22 (0.81–6.10)	.121			1.38 (0.56–3.44)	.485		
**ECOG (1 vs. 0)**	0.90 (0.57–1.44)	.670			0.90 (0.56–1.46)	.679		
**Vascular invasion (yes vs. no)**	1.29 (0.80–2.08)	.303	1.24 (0.72–2.15)	.442	1.28 (0.78–2.11)	.330	1.16 (0.65–2.05)	.617
**Extrahepatic metastasis (yes vs. no)**	0.97 (0.57–1.66)	.924	0.98 (0.55–1.75)	.955	0.94 (0.54–1.62)	.819	0.86 (0.46–1.58)	.621
**Barcelona staging (BCLC‐C vs. BCLC‐B)**	0.64 (0.34–1.19)	.156	0.61 (0.30–1.26)	.183	0.74 (0.39–1.37)	.334	0.43 (0.20–0.92)	.030
**Treatment lines (2 vs. 1)**	1.13 (0.71–1.80)	.593			0.90 (0.56–1.45)	.665		
**Albumin**	1.01 (0.96–1.07)	.648			1.00 (0.95–1.05)	.956		
**Total bilirubin**	1.02 (0.98–1.07)	.327			1.03 (0.98–1.08)	.244		
**Tumour burden**	1.00 (1.00–1.01)	.819			1.00 (1.00–1.01)	.753		
**α‐fetoprotein concentration (>400 vs. ≤400)**	1.10 (0.69–1.76)	.674	0.88 (0.52–1.47)	.624	1.76 (1.09–2.86)	.021	1.39 (0.82–2.37)	.226
**LDH(>174.55 vs. ≤174.55)**	1.82 (0.96–3.45)	.066			2.22 (1.10–4.48)	.026	1.46 (0.68–3.15)	.331
**NLR(>1.49 vs. ≤1.49)**	2.44 (0.98–6.09)	.055			3.13 (1.14–8.61)	.027	2.69 (0.92–7.86)	.070
**PLR(>124.95 vs. ≤124.95)**	1.22 (0.77–1.91)	.399			1.42 (0.89–2.28)	.145	1.32 (0.77–2.27)	.316
**bTMB (≤4 vs. >4)**	0.67 (0.43–1.05)	.080	0.77 (0.47–1.27)	.308	0.57 (0.36–0.92)	.020	0.71 (0.42–1.19)	.192
**bMSAF (≤0.027 vs. >0.027)**	0.46 (0.27–0.79)	.005	0.49 (0.26–0.90)	.023	0.41 (0.23–0.73)	.002	0.61 (0.31–1.18)	.141

ECOG, Eastern Cooperative Oncology; BCLC, Barcelona Clinic Liver Cancer; LDH, lactate dehydrogenase; NLR, neutrophil‐to‐lymphocyte ratio; PLR, platelet‐lymphocyte ratio; bTMB, blood‐based tumour mutation burden; bMSAF, blood‐based maximum somatic allele frequency.

Mutation landscape derived from blood samples confirmed frequent somatic mutations (Supplementary Figure S9A). Only *NCOR1* mutations were significantly associated with DCR (Supplementary Figure S9B). Patients with *RB1*, *ROS1*, *PBRM1*, *NCOR1*, *KEAP1* or *AR* mutations had worse PFS or OS (Supplementary Figure S9C), and these mutations had no remarkable correlation with bMSAF (Supplementary Figure S9D), indicating that impact of bMSAF on survival was independent of these prognostic‐related genes. Meanwhile, 10 canonical pathways were enriched, in which mutations in Cell Cycle and NRF2 pathways were associated with poor survival (Supplementary Figure S10A and B), but non‐significant trend in ORR and DCR (Supplementary Figure S10C).

In conclusion, bMSAF is more valuable baseline circulating marker than bTMB for predicting prognosis in advanced HCC patients treated with camrelizumab and apatinib combination therapy.

## CONFLICT OF INTEREST

The authors declare no potential conflict of interest.

## Supporting information

Supporting Information

Supporting Information

Supporting Information

Supporting Information

Supporting Information

Supporting Information

Supporting Information

Supporting Information

Supporting Information

Supporting Information

Supporting Information

Supporting Information
