# Supplementary figures and images for: bMSAF is a prognostic predictor for advanced hepatocellular carcinoma patients treated with immune checkpoint inhibitor camrelizumab and anti‐angiogenic agent apatinib combination therapy
Source: Clin Transl Med. 2022 Oct 17;12(10):e1086. doi: 10.1002/ctm2.1086 (PMC9574487; doi:10.1002/ctm2.1086)

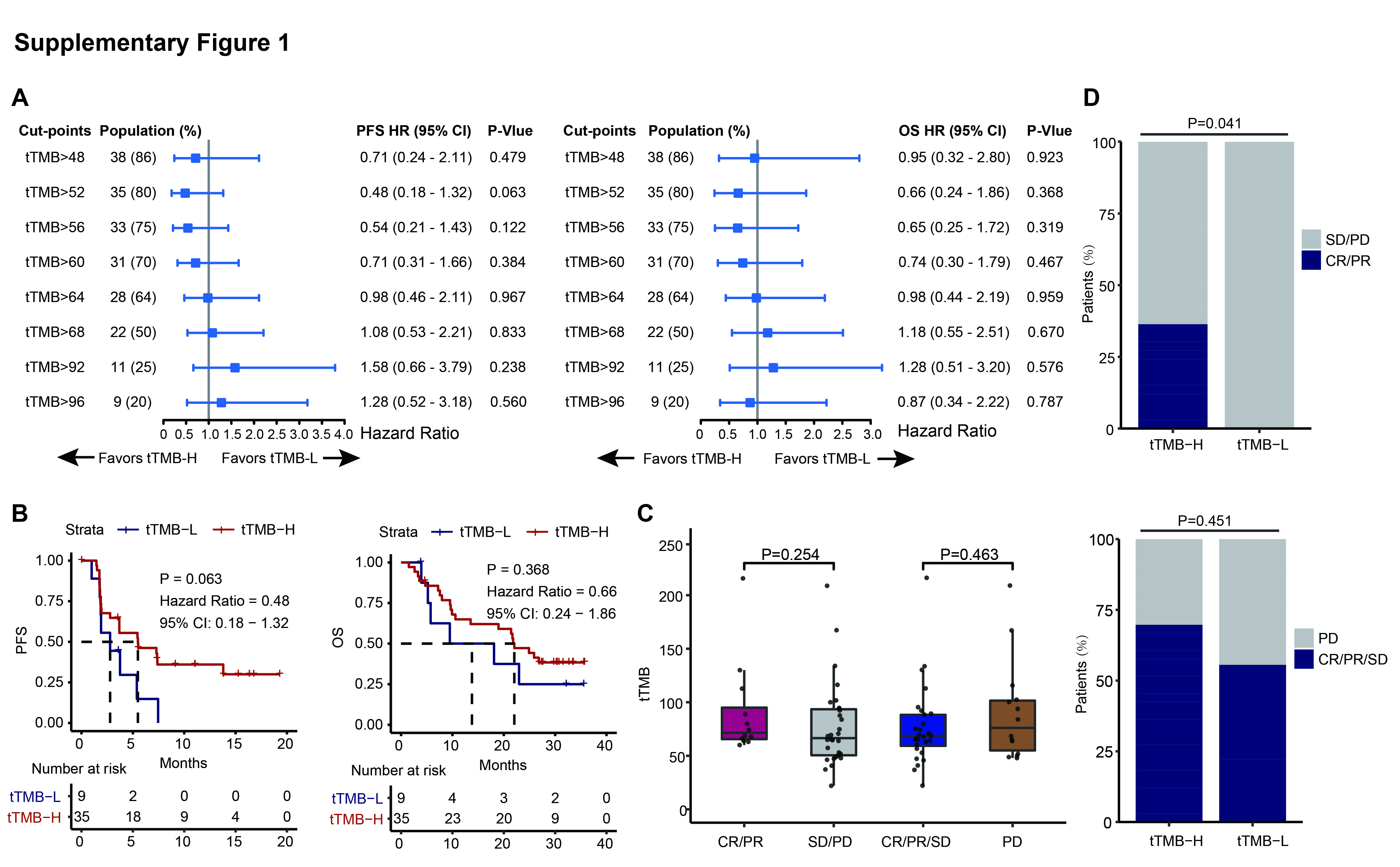

Supplement: Supplementary file 1 — Supporting Information [file CTM2-12-e1086-s008.jpg]

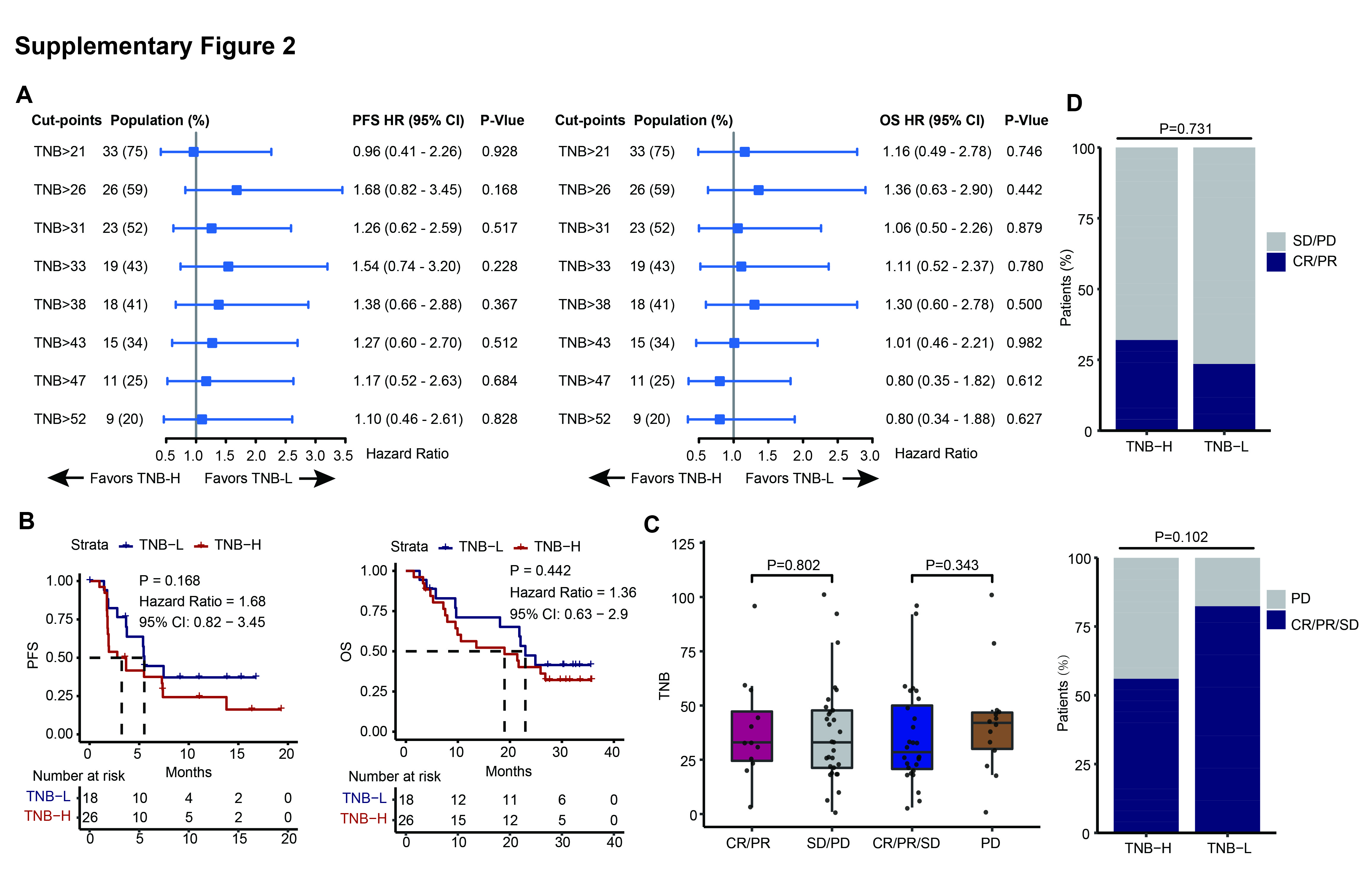

Supplement: Supplementary file 2 — Supporting Information [file CTM2-12-e1086-s011.jpg]

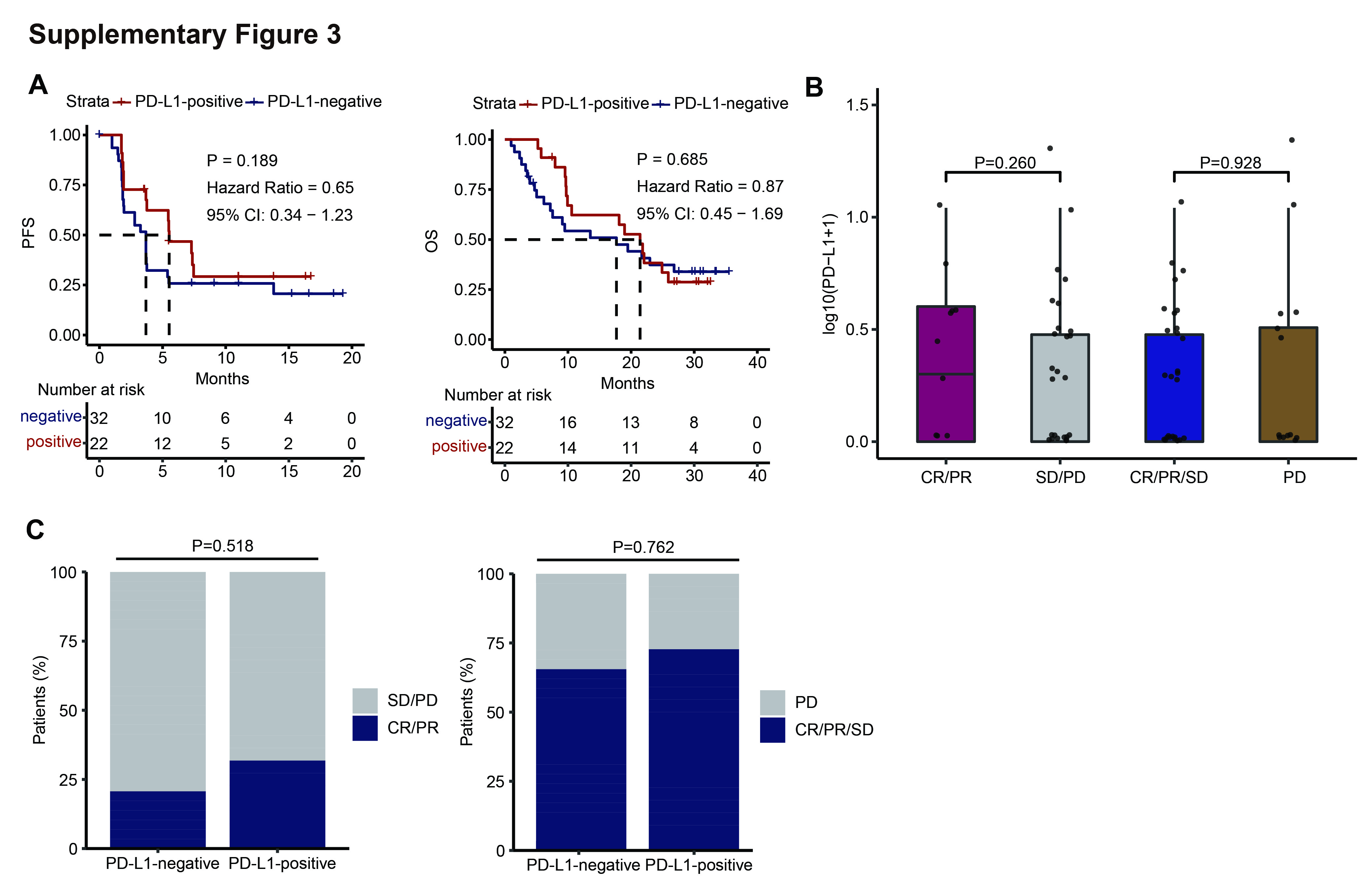

Supplement: Supplementary file 3 — Supporting Information [file CTM2-12-e1086-s009.jpg]

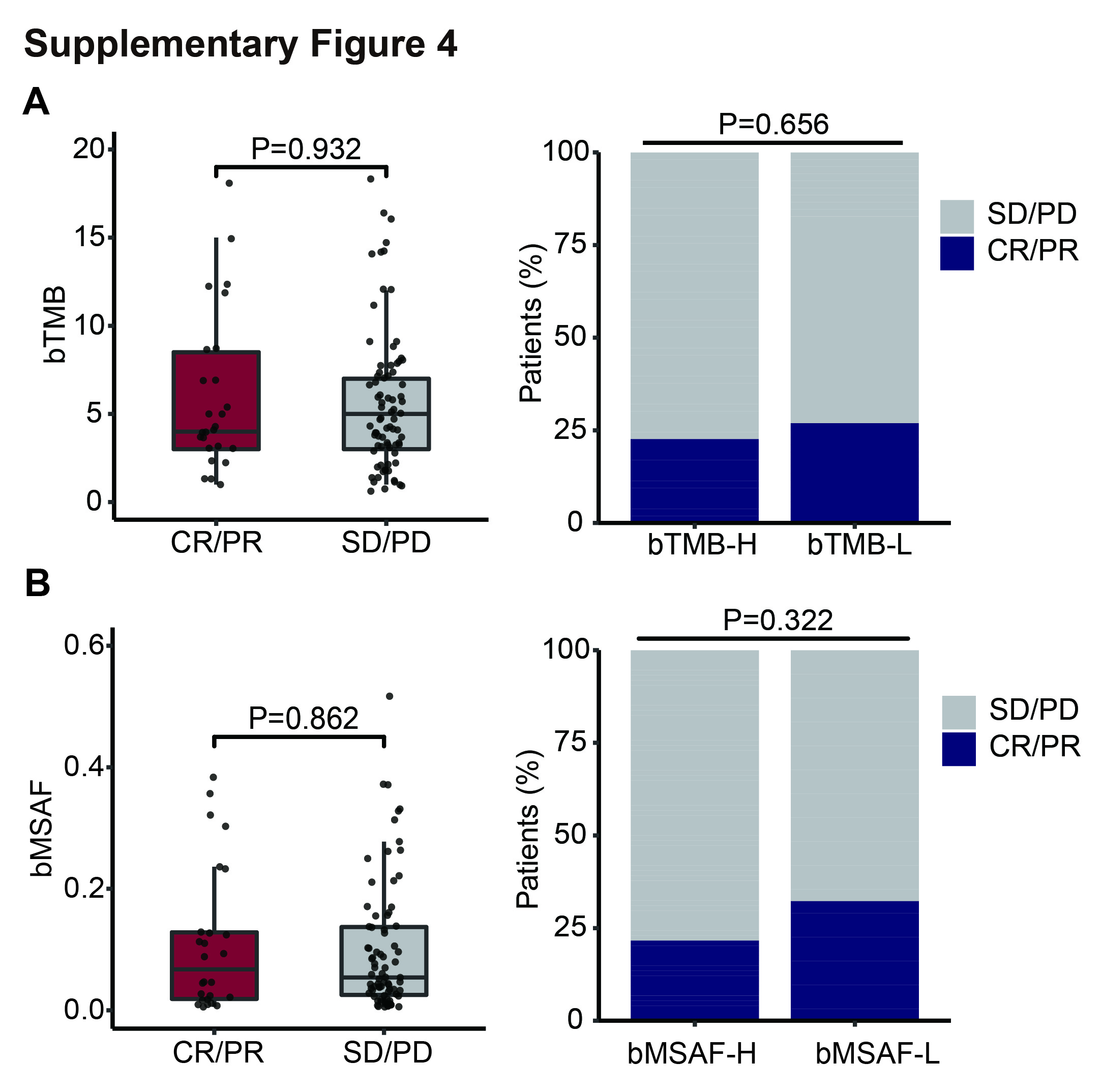

Supplement: Supplementary file 4 — Supporting Information [file CTM2-12-e1086-s004.jpg]

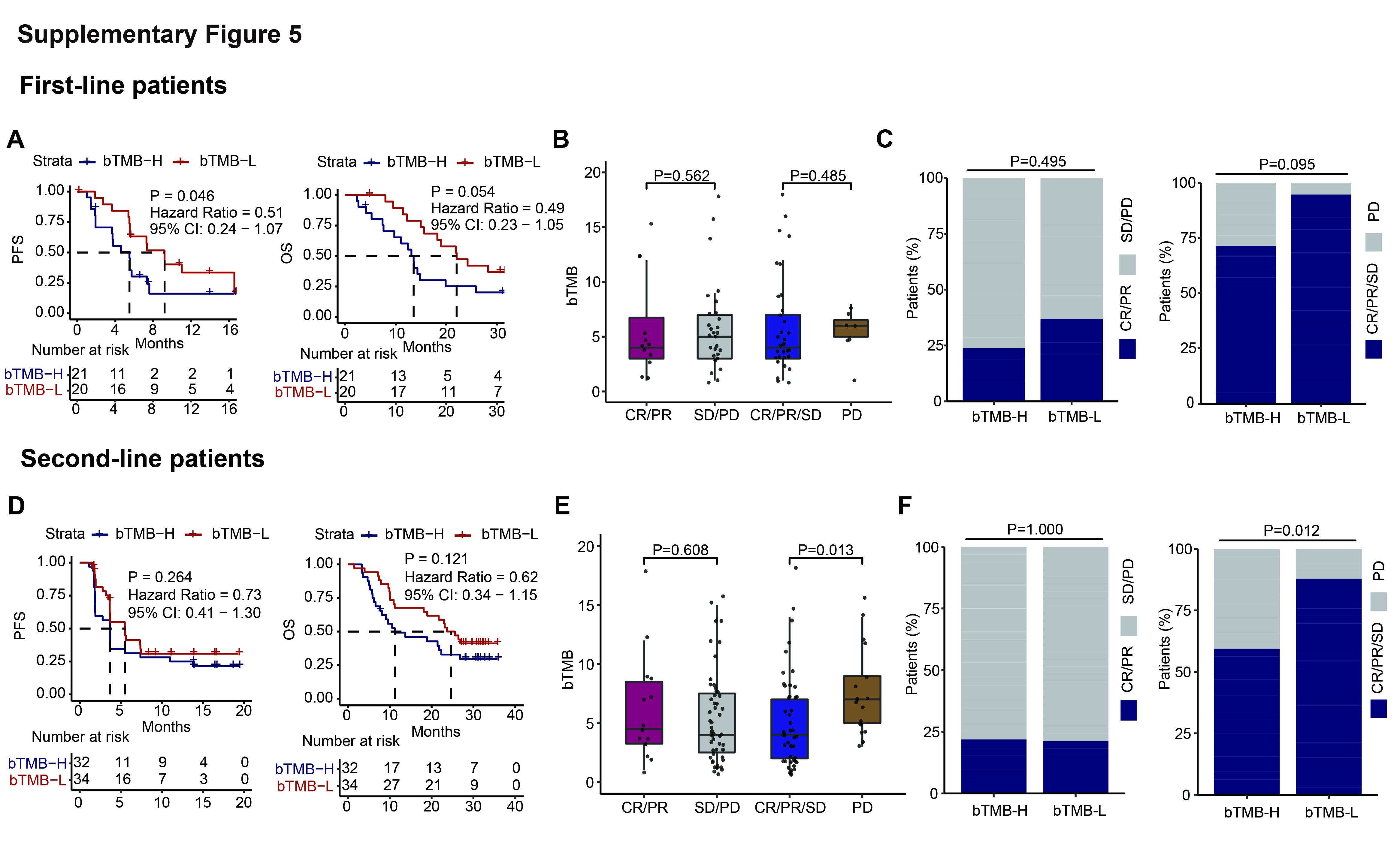

Supplement: Supplementary file 5 — Supporting Information [file CTM2-12-e1086-s003.jpg]

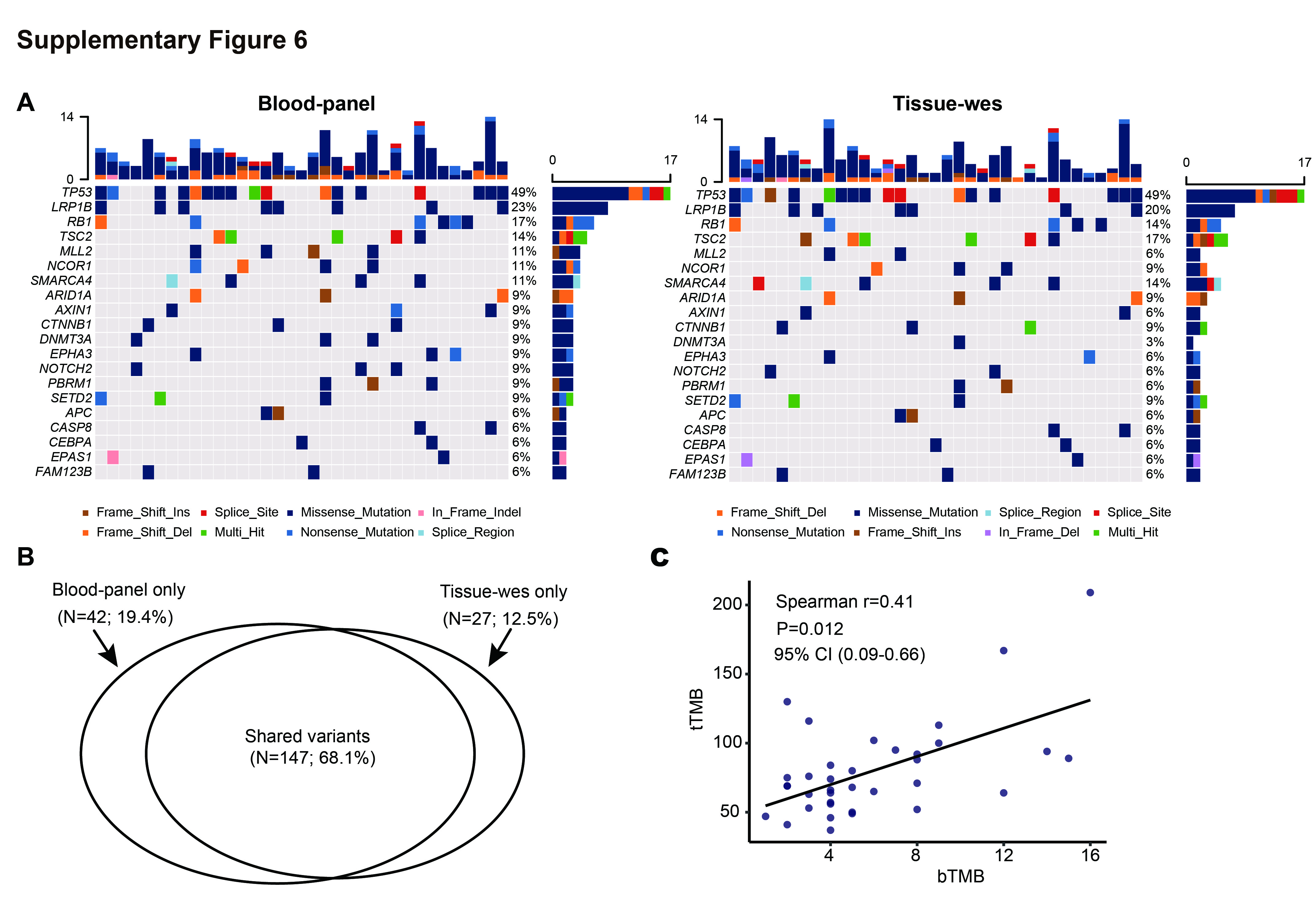

Supplement: Supplementary file 6 — Supporting Information [file CTM2-12-e1086-s006.jpg]

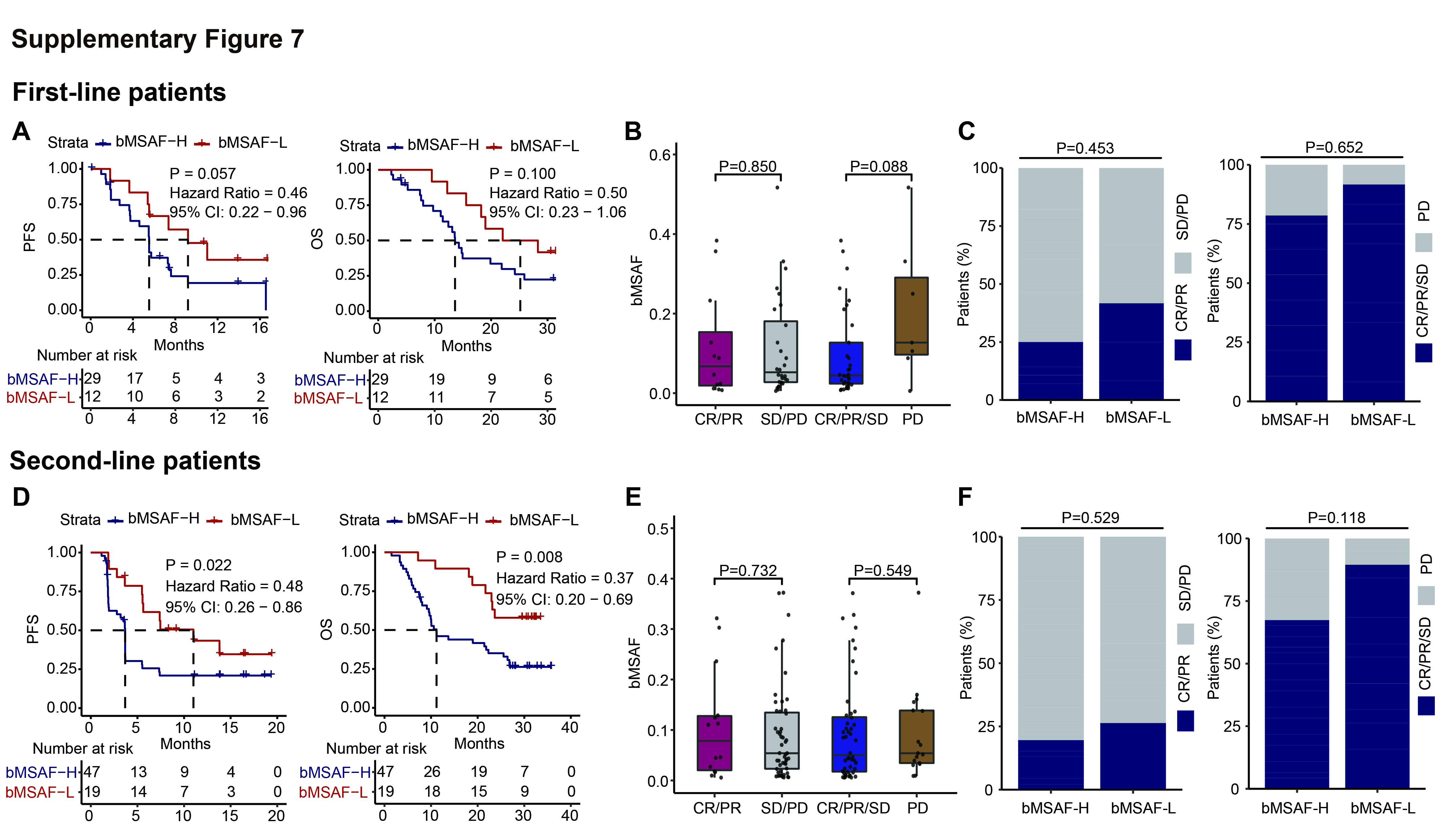

Supplement: Supplementary file 7 — Supporting Information [file CTM2-12-e1086-s001.jpg]

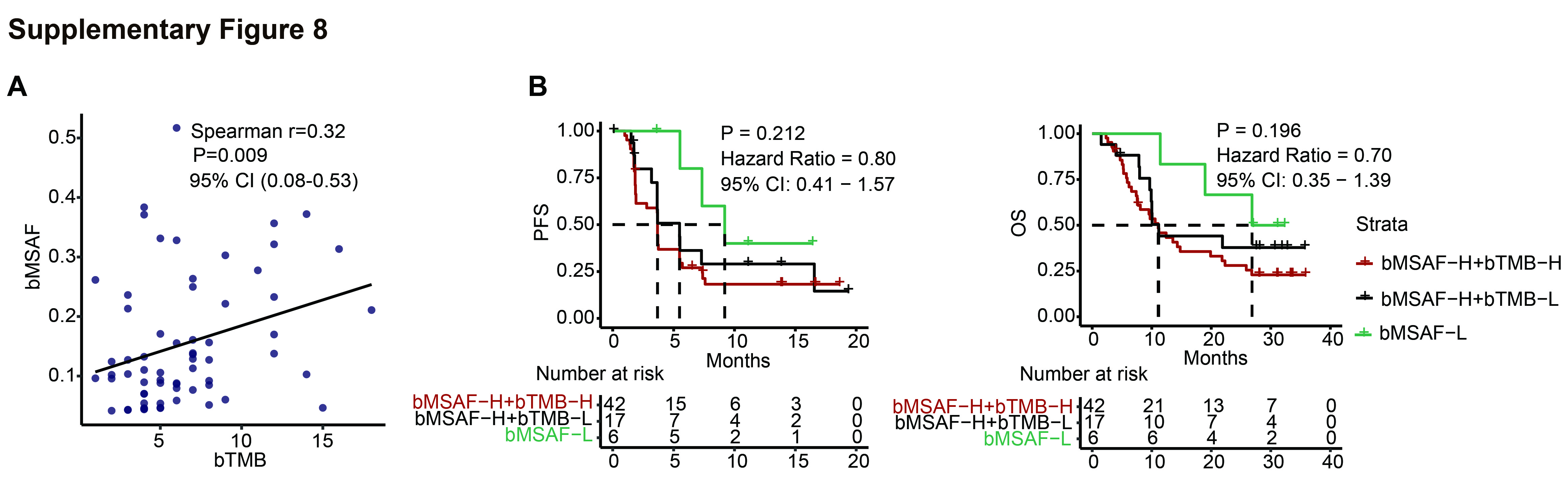

Supplement: Supplementary file 8 — Supporting Information [file CTM2-12-e1086-s007.jpg]

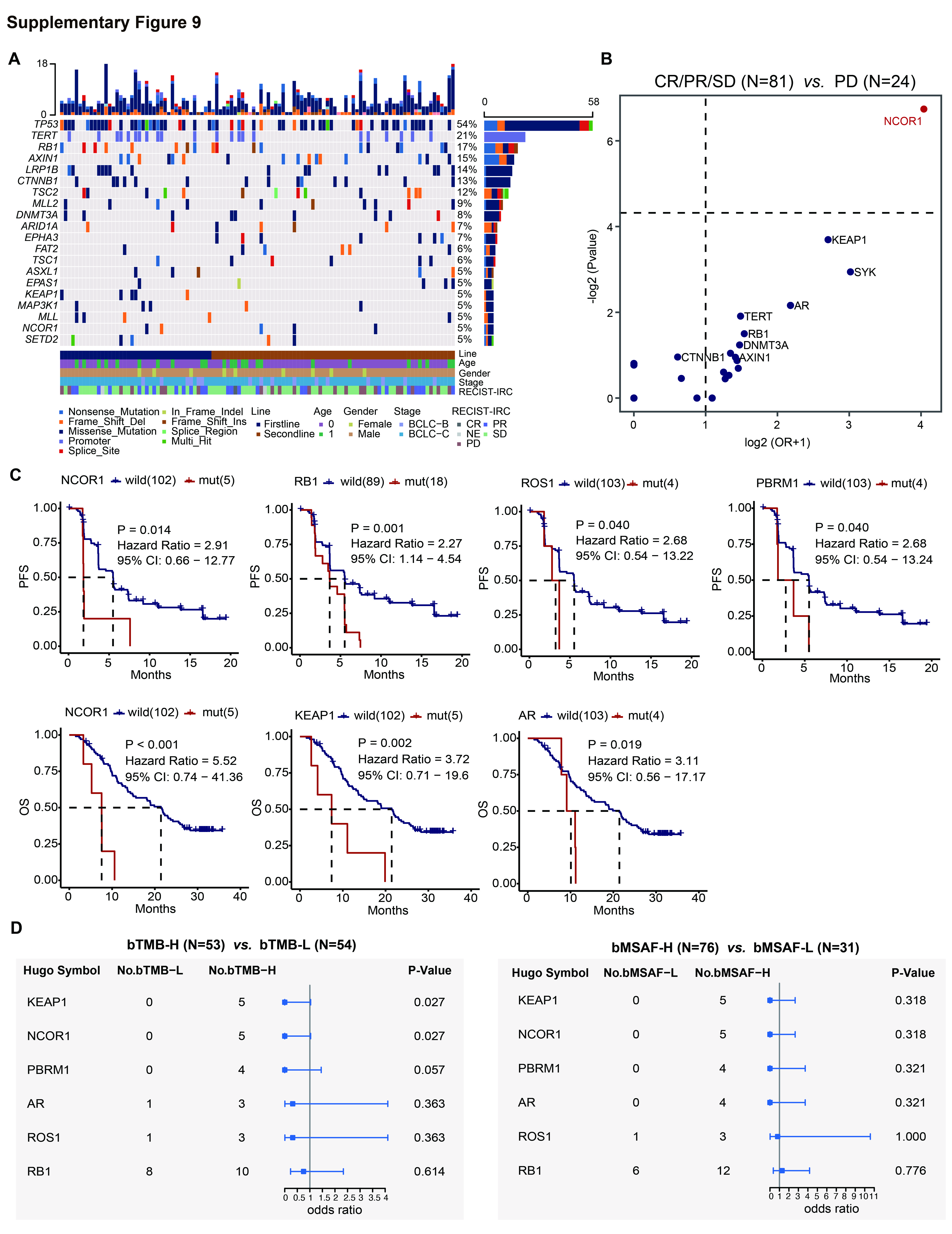

Supplement: Supplementary file 9 — Supporting Information [file CTM2-12-e1086-s012.jpg]

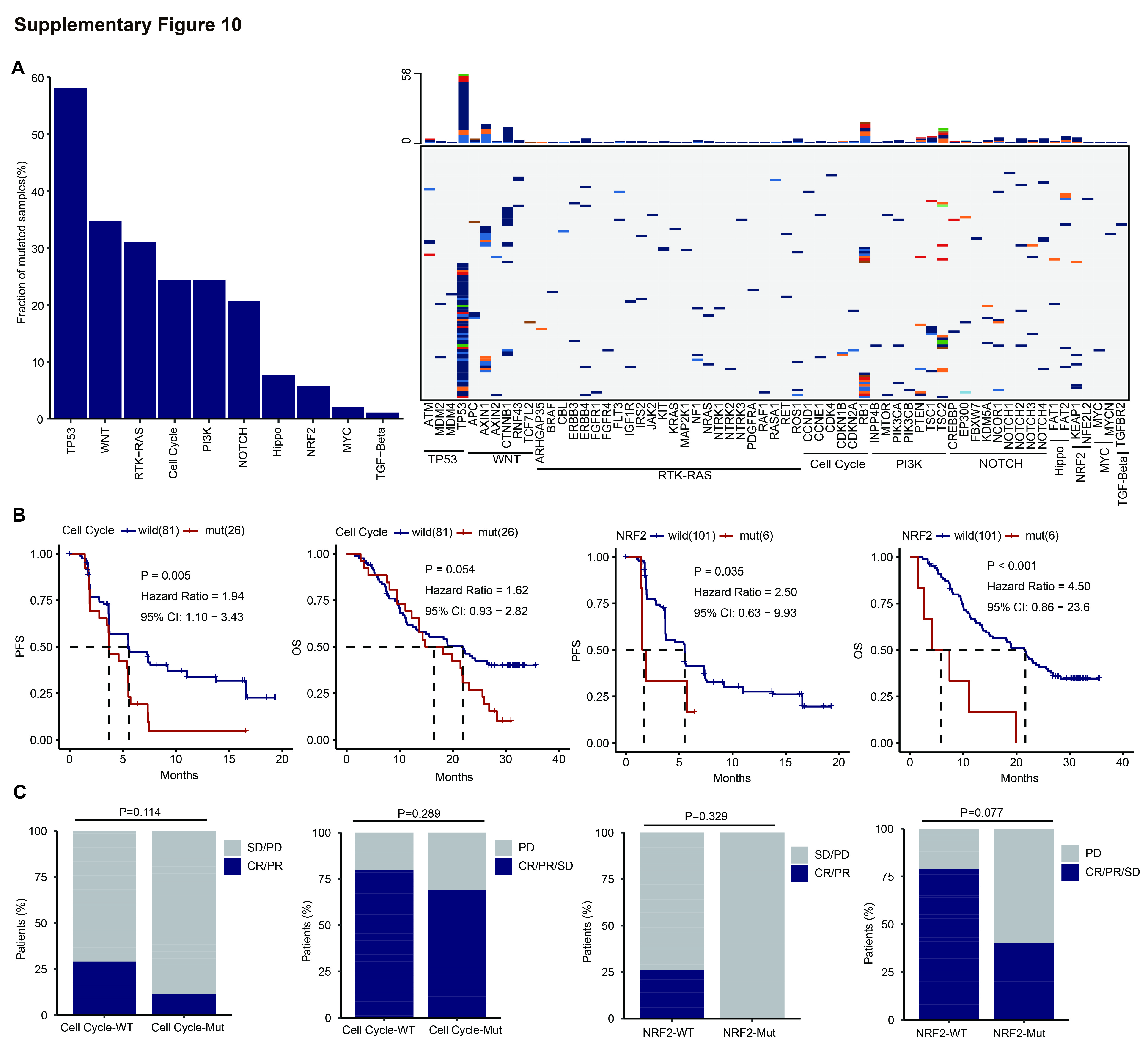

Supplement: Supplementary file 10 — Supporting Information [file CTM2-12-e1086-s005.jpg]
