# Supplementary material for: bMSAF is a prognostic predictor for advanced hepatocellular carcinoma patients treated with immune checkpoint inhibitor camrelizumab and anti‐angiogenic agent apatinib combination therapy
Source: Clin Transl Med. 2022 Oct 17;12(10):e1086. doi: 10.1002/ctm2.1086 (PMC9574487; doi:10.1002/ctm2.1086)
Supplement: Supplementary file 11 — Supporting Information [file CTM2-12-e1086-s002.docx]

**Supplementary information**

**Materials and Methods**

**Patients**

This study included 118 patients with advanced HCC who were enrolled in a non-randomized, multicenter, open-label, Phase II trial to receive intravenous camrelizumab plus oral apatinib^1^. From these patients, 118 blood samples and 55 tissue samples were collected at baseline. Ten milliliter of peripheral blood was collected from each patient before treatment. Tumor tissues were collected and fixed in formalin embedded in paraffin (FFPE). The study was conducted in accordance with the International Conference on Good Clinical Practice Standards and the Declaration of Helsinki and were approved by each institution’s ethical review board. All patients provided written informed consent. Clinical trial design and outcomes about this study have been reported in the Phase II RESCUE trial^1^.

**Sample exclusion**

Of the total 118 blood samples collected, 107 were available for analysis, as 2 were excluded for insufficient concentration of DNA extraction or library construction, 8 were excluded due to no mutations were detected, and 1 was excluded due to variant allele frequency (VAF) of the gene was less than 0.5%.

Of the total 55 tissue samples, in which only 49 tissue samples were sequenced and provided paired blood samples. 44 were sent for biomarker analysis, as four were excluded for insufficient concentration of DNA extraction or library construction, and one was excluded due to no mutations were detected.

After exclusion of the samples described above, 36 paired tissue-blood samples were available for further analysis, in addition to individual tissue and blood samples.

**Clinical efficacy assessments**

Due to the primary endpoint of previous phase II RESCUE study was assessed by an independent review committee (IRC) as per RECIST v1.1 guidelines,^1^ thus response assessment was still used IRC per RECIST v1.1 in this study. Objective response rate (ORR) was defined as the proportion of patients who achieved confirmed complete response (CR) or partial response (PR). The disease control rate (DCR) was defined as the proportion of patients who achieved CR, PR, or stable disease (SD). Progressive disease was abbreviated as PD. PFS was the time from the first dose of study medication to radiographic disease progression or death. OS was the time from the first dose of study medication to death due to any cause. After Phase II RESCUE study, the data of RECIST assessment, PFS and OS were updated, and the updated efficacy and survival data were used in this study.

**Target panel sequencing**

To detect circulating tumor DNA (ctDNA), circulating cell-free DNA (cfDNA) was isolated from 0.6–1.8 mL plasma using the QIAsymphony circulating DNA kit (Qiagen, Hilden, Germany). Germline genomic DNA (gDNA) was extracted from peripheral blood lymphocytes using the DNeasy Blood & Tissue Kit (Qiagen). DNA concentration was measured using a Qubit fluorometer and the Qubit dsDNA High-Sensitivity (HS) Assay Kit (Invitrogen, Carlsbad, CA, USA). Size distribution of the cfDNA was assessed using an Agilent 2100 BioAnalyzer and the DNA HS kit (Agilent Technologies, Santa Clara, CA, USA). All DNA extractions were performed according to the manufacturers’ instructions.

Before library construction, 1.0 µg gDNA was sheared to 200-250 bp fragments using a Covaris S2 ultrasonicator (Covaris, Woburn, MA, USA). 80 ng blood cfDNA and fragmented gDNA were was added to Illumina indexed adapters for library construction using the KAPA Library Preparation Kit (Kapa Biosystems, Wilmington, MA, USA). Custom-designed probes, which covered 1.6 Mb regions for 1021 cancer-related genes were used for DNA capture. All genes and coordinates of selected regions were as described previously^2^. Sequencing was performed using the NovaSeq 6000 or NextSeq CN500 Sequencing System (Illumina, San Diego, CA, USA) with 2×100 or 75-bp paired-end reads.

Sequence analysis was performed using BWA (version 0.7.12-r1039) to align clean reads to the reference human genome (hg19). Single nucleotide variants (SNVs) and small insertions and deletions were called using MuTect (version 1.1.4). Somatic mutations were identified by VAF≥ 0.5% and at least 5 high quality reads (Phred score ≥30, mapping quality ≥30, and without paired-end reads bias). Mutations were annotated with genes using ANNOVAR^3^ software.

**Whole-exome sequencing (WES)**

Tissue DNA was extracted using the Maxwell^®^ 16 FFPE Plus LEV DNA Purification Kit (Promega, Madison, WI, USA). Before library construction, 1.0 μg each of gDNA and tissue DNA was sheared to 200-250 bp fragments with a Covaris S2 ultrasonicator (Covaris, Woburn, MA, USA). Indexed libraries were prepared using the KAPA Library Preparation Kit (Kapa Biosystems) and then were hybridized to SeqCap EZ Exome 64M (Roche NimbleGen, Madison, WI, USA) according to the manufacturer's instructions. Sequencing was carried out with the NextSeq CN500 or NovaSeq 6000 Sequencing System with 2×75 or 100 or 150-bp paired-end reads. The terminal adaptor sequences and low-quality reads were removed from the raw data. BWA (version 0.7.12-r1039) was employed to align the clean reads to the reference human genome (hg19). Picard (version 1.98) was used to mark PCR duplicates. Realignment and recalibration were performed using GATK (version 3.4-46-gbc02625). Somatic mutations were called as previously reported^4^.

**TMB analysis**

bTMB and tTMB were defined as the number of non-synonymous mutations detected at an allele frequency of ≥0.5% or 5%, respectively.

**TNB analysis**

Germline HLA-I genotyping from normal PBMC sequencing data was performed using OptiType^5^. Then, somatic mutations, including non-synonymous single base substitutions, insertions, and deletions, were used to comprehensively assess peptides 8 to 11 amino acids in length at every position surrounding a somatic mutation. The major histocompatibility complex (MHC) class I binding potential of each somatic and wild (WT) peptide was predicted by NetMHCpan 4.0. The number of neoantigens with a high MHC class I binding affinity (IC_50_ value <500 nM) of each sample was counted as TNB for further analysis.

**PD-L1 expression**

PD-L1 expression was assessed by immunohistochemistry with the PD-L1 IHC 22C3 pharmDx kit (Agilent Technologies). Expression of PD-L1 was determined by the Tumor Proportion Score (TPS), which is defined as the percentage of viable tumor cells showing partial or complete membrane staining. The detailed protocol was described in previous study^1^.

**Enrichment of cancer-related pathways**

The enrichment of known Oncogenic Signaling Pathways^6^ in TCGA cohorts was achieved though “OncogenicPathways” function in maftools R packages. The gene mutation file in MAF format was used as an input.

**Neutrophil-lymphocyte ratio and platelet-lymphocyte ratio**

The neutrophil-lymphocyte ratio (NLR) was calculated by dividing the absolute number of neutrophils by the absolute number of lymphocytes from a simple blood test. The platelet-to-lymphocyte ratio (PLR) was defined as the ratio of absolute platelet count divided by the absolute lymphocyte count. The optimal cutoff points of NLR and PLR were determined by Youden index (Youden index equals to sensitivity plus specificity minus 100%).

**Statistical analysis**

Survival analysis was performed using the Kaplan-Meier method by the log-rank test. The function of “surv_cutpoint” in the survminer R package was used to determine optimal cutoff value of continuous variables for survival analyses according to PFS, using the maximally selected rank statistics as implemented in the maxstat R package^7^. Correlations were calculated using Spearman rank correlation testing. Differences of continuous variables between two groups were examined by Wilcoxon rank-sum test. Categorical variables related to therapeutic outcome were analyzed using Fisher’s exact test. Univariate and multivariable Cox regression were used to study the association between different variables and PFS or OS, and the results are presented as hazard ratios (HRs) and their 95% confidence intervals (CIs). All statistical tests were two-sided, and results with P <0.05 were considered as statistically significant. All analyses and figures were performed or generated with R 4.0.3.

**References**

1. Xu J, Shen J, Gu S, et al. Camrelizumab in Combination with Apatinib in Patients with Advanced Hepatocellular Carcinoma (RESCUE): A Nonrandomized, Open-label, Phase II Trial. *Clin Cancer Res.* 2021;27(4):1003-1011.

2. Liu X, Wang F, Xu C, et al. Genomic origin and intratumor heterogeneity revealed by sequencing on carcinomatous and sarcomatous components of pulmonary sarcomatoid carcinoma. *Oncogene.* 2021;40(4):821-832.

3. Wang K, Li M, Hakonarson H. ANNOVAR: functional annotation of genetic variants from high-throughput sequencing data. *Nucleic Acids Res.* 2010;38(16):e164.

4. Wu K, Zhang X, Li F, et al. Frequent alterations in cytoskeleton remodelling genes in primary and metastatic lung adenocarcinomas. *Nat Commun.* 2015;6:10131.

5. Chowell D, Morris L, Grigg C, et al. Patient HLA class I genotype influences cancer response to checkpoint blockade immunotherapy. *Science (New York, NY).* 2018;359(6375):582-587.

6. Sanchez-Vega F, Mina M, Armenia J, et al. Oncogenic Signaling Pathways in The Cancer Genome Atlas. *Cell.* 2018;173(2):321-337.e310.

7. Hothorn T. Maxstat: maximally selected rank statistics. R package version 0.7-12, URL <http://CRAN>. R-project. org/package= maxstat; 2007.

**Supplementary Figure Legends:**

**Figure S1. Assessment of the predictive effect of tTMB on HCC patients treated with ICI combined with anti-angiogenic therapy.** (A) Forest plots of hazard ratios (HRs) of progression-free survival (PFS) and overall survival (OS) comparing patients at varying tTMB cutpoints. (B) Kaplan-Meier analysis of PFS and OS in patients with tTMB-L (tTMB ≤52) and tTMB-H (tTMB >52). (C) tTMB in patients with complete response (CR)/partial response (PR) (n=12) versus those with stable disease (SD)/progressive disease (PD) (n =30) (median 71.5 versus 66.5 mutations, Wilcoxon P = 0.254) and tTMB in patients with CR/PR/SD (n=28) vs. PD (n=14) (median: 68.5 vs. 76 mutations, Wilcoxon P =0.463). (D) Objective response and disease control in patients with tTMB-H versus those with tTMB-L (ORR: 36% versus 0, Fisher’s exact P= 0.041; DCR: 70% versus 56%, Fisher’s exact P = 0.451).

**Figure S2. TNB could not distinguish benefit patients.** (A) Forest plots of HRs of PFS and OS comparing patients at varying TNB cutpoints. (B) Kaplan-Meier analysis of PFS and OS in patients with TNB-L (TNB ≤26) and TNB-H (TNB >26). (C) TNB in patients with complete response (CR)/partial response (PR) (n=12) versus those with stable disease (SD)/progressive disease (PD) (n =30) (median 33 versus 33, Wilcoxon P = 0.802) and TNB in patients with CR/PR/SD (n=28) vs. PD (n=14) (median: 28.5 vs. 42, Wilcoxon P =0.343). (D) Objective response and disease control in patients with TNB-H versus those with TNB-L (ORR: 32% versus 24%, Fisher’s exact P= 0.731; DCR: 56% versus 82%, Fisher’s exact P = 0.102).

**Figure S3. PD-L1 expression is not a good predictor of treatment response.** (A) Kaplan-Meier analysis of PFS and OS in patients who are PD-L1-positive or PD-L1-negative. (B) PD-L1 expression in patients with CR/PR (n=13) versus those with SD/PD (n =38) (median 1 versus 0, Wilcoxon P = 0.260) and PD-L1 in patients with CR/PR/SD (n=35) vs. PD (n=16) (median: 0 vs. 0, Wilcoxon P =0.928). (C) Objective response and disease control in patients with PD-L1-negative versus those with PD-L1-positive (ORR: 21% versus 32%, Fisher’s exact P= 0.518; DCR: 66% versus 73%, Fisher’s exact P = 0.762).

**Figure S4. Objective response of bTMB and bMSAF in patients with HCC treated with camrelizumab plus apatinib.** (A) Left: bTMB in patients with CR/PR (n=26) versus those with SD/PD (n=79) (median: 4 vs. 5, Wilcoxon P=0.932). Right: objective response in patients with bTMB-H versus those with bTMB-L (ORR: 23% versus 27%, Fisher’s exact P = 0.656). (B) Left: bMSAF in patients with CR/PR (n=26) versus those with SD/PD (n=79) (median: 0.067 vs. 0.054, Wilcoxon P=0.862). Right: objective response in patients with bMSAF-H versus those with bMSAF-L (ORR: 22% versus 32%, Fisher’s exact P = 0.322).

**Figure S5. Effects of bTMB on survival and efficacy of camrelizumab combined with apatinib in first- and second-line patients.** (A) Kaplan-Meier analysis of PFS and OS in first-line patients with bTMB-L (bTMB ≤4) and bTMB-H (bTMB >4). (B) bTMB in first-line patients with CR/PR (n=12) versus those with SD/PD (n =28) (median: 4 versus 5 mutations, Wilcoxon P = 0.562) and bTMB in first-line patients with CR/PR/SD (n=33) vs. PD (n=7) (median: 4 vs. 6 mutations, Wilcoxon P =0.485). (C) Objective response and disease control in first-line patients with bTMB-H versus those with bTMB-L (ORR: 24% versus 37%, Fisher’s exact P= 0.495; DCR: 71% versus 95%, Fisher’s exact P = 0.095). (D) Kaplan-Meier analysis of PFS and OS in second-line patients with bTMB-L (bTMB ≤4) and bTMB-H (bTMB >4). (E) bTMB in second-line patients with CR/PR (n=14) versus those with SD/PD (n =51) (median: 4.5 versus 4 mutations, Wilcoxon P = 0.608) and bTMB in second-line patients with CR/PR/SD (n=48) vs. PD (n=17) (median: 4 vs. 7 mutations, Wilcoxon P =0.013). (F) Objective response and disease control in second-line patients with bTMB-H versus those with bTMB-L (ORR: 22% versus 21%, Fisher’s exact P= 1.000; DCR: 59% versus 88%, Fisher’s exact P = 0.012).

**Figure S6. Concordance of genetic profiles in paired baseline blood and tissue samples.** (A) The top 20 genes in terms of mutation frequency genes are represented in the oncoprint. Baseline blood samples sequenced by panel 1021 are on the left, and paired tissue samples sequenced by whole exon sequencing (WES) are on the right. (B) A Venn diagram shows the overlapping (68.1%) and mutually exclusive (12.5% tissue only; 19.4% blood only) variants. (C) Pairwise comparison of tTMB and bTMB from patients (N = 36) sequenced by both targeted panel and WES (Spearman r= 0.41; 95% CI: 0.09–0.66; P=0.012). The number of detected mutations is represented on each axis: for the bTMB mutations, the counts include non-synonymous mutations at allele frequencies of ≥0.5%. for the tTMB mutations, the counts include non-synonymous mutations at allele frequencies of ≥5%.

**Figure S7. Effects of bMSAF on survival and efficacy of camrelizumab combined with apatinib in first- and second-line patients.** (A) Kaplan-Meier analysis of PFS and OS in first-line patients with bMSAF-L (bMSAF ≤0.027) and bMSAF-H (bMSAF >0.027). (B) bMSAF in first-line patients with CR/PR (n=12) versus those with SD/PD (n =28) (median 0.067 versus 0.053, Wilcoxon P = 0.850) and bMSAF in first-line patients with CR/PR/SD (n=33) vs. PD (n=7) (median: 0.044 vs. 0.127, Wilcoxon P =0.088). (C) Objective response and disease control in first-line patients with bMSAF-H versus those with bMSAF-L (ORR: 25% versus 42%, Fisher’s exact P= 0.453; DCR: 79% versus 92%, Fisher’s exact P = 0.652). (D) Kaplan-Meier analysis of PFS and OS in second-line patients with bMSAF-L (bMSAF ≤0.027) and bMSAF-H (bMSAF >0.027). (E) bMSAF in second-line patients with CR/PR (n=14) versus those with SD/PD (n =51) (median 0.078 versus 0.054, Wilcoxon P = 0.732) and bMSAF in second-line patients with CR/PR/SD (n=48) vs. PD (n=17) (median: 0.050 vs. 0.054, Wilcoxon P =0.549). (F) Objective response and disease control in second-line patients with bMSAF-H versus those with bMSAF-L (ORR: 20% versus 26%, Fisher’s exact P= 0.529; DCR: 67% versus 89%, Fisher’s exact P = 0.118).

**Figure S8. bMSAF more precisely predicted clinical outcomes.** (A) Poor correlation between bTMB and bMSAF in patients with ctDNA MSAF >4% (Spearman r=0.32, P=0.009). (B) PFS and OS among patients with bMSAF-H+bTMB-H, bMSAF-H+bTMB-L, and bMSAF-L status.

**Figure S9.** **Molecular characteristics associated with prognosis.** (A) Somatic gene mutational landscape of 107 blood samples. The top 20 genes in terms of mutation frequency genes are represented in the oncoprint, with the distribution of clinical characteristics and response of each patient shown at the bottom. (B) Plot showing log2 (OR+1) (x axis) vs. -log2 (P value) (y axis) for a comparison of gene mutation frequency in CR/PR/SD versus PD groups (P value using Fisher’s exact test). Genes with P <0.05 are colored in red. OR: odd ratio. (C) Genes related to prognosis were identified from blood samples. Genes that have a significant association with PFS are shown at the top. Genes at the bottom are remarkably associated with OS. (D) Forest plots of odds ratio (OR) of bTMB-H and bTMB-L or bMSAF-H and bMSAF-L comparing patients at varying gene mutations. Fisher’s exact test is used to analyze significance.

**Figure S10. The enrichment of cancer-related pathways.** (A) The mutation frequencies of 10 enriched cancer related pathways and mutation genes in each pathway are shown. (B) Kaplan-Meier analysis of PFS and OS in patients with Cell Cycle or NRF2 pathway. (C) ORR and DCR in patients with Cell Cycle-WT versus Cell Cycle-Mut (ORR: 29% vs. 12%, Fisher’s exact P=0.114; DCR: 80% vs. 69%, Fisher’s exact P=0.289) or NRF2-WT versus NRF2-Mut (ORR: 26% vs. 0, Fisher’s exact P=0.329; DCR: 79% vs. 40%, Fisher’s exact P=0.077).

**Supplementary Table Legends:**

**Table S1. Clinical and outcomes characteristics of individual patients.**

**Table S2. Baseline clinical characteristics in RESCUE and present studies.**

**Table S3. Quality control information of 44 liver cancer samples sequenced by WES.**

**Table S4. Genomic characteristics of 44 liver cancer tissue samples.**

**Table S5. The analysis of tumor neoantigen burden for 44 liver tumor tissue samples.**

**Table S6. Quality control information of 107 liver blood samples sequenced by 1021 targeted panel.**

**Table S7. Genomic characteristics of ctDNA from 107 baseline blood samples.**

**Table S8. Baseline clinical characteristics and association with bTMB and bMSAF.**

**Table S9. Univariate and multivariate analyses of bMSAF on PFS and OS using important clinical characteristics and bTMB as covariates in first-line patients.**

**Table S10. Univariate and multivariate analyses of bMSAF on PFS and OS using important clinical characteristics and bTMB as covariates in second-line patients.**
